# Editorial: Tailoring NK Cell Receptor–Ligand Interactions: An Art in Evolution

**DOI:** 10.3389/fimmu.2018.00351

**Published:** 2018-02-27

**Authors:** Ulrike Koehl, Antoine Toubert, Gianfranco Pittari

**Affiliations:** ^1^Hannover Medical School, Institute of Cellular Therapeutics, Hannover, Germany; ^2^Institute of Clinical Immunology, University Hospital and Medical Faculty, University of Leipzig, Leipzig, Germany; ^3^Fraunhofer Institute for Cell Therapy and Immunology, Leipzig, Germany; ^4^Institut National de la Santé et de la Recherche Médicale (INSERM) U1160; Université Paris Diderot, Sorbonne Paris Cité, Paris, France; ^5^Laboratoire d’Immunologie et Histocompatibilité, Hópital Saint-Louis, Paris, France; ^6^Department of Medical Oncology, National Center for Cancer Care and Research, Hamad Medical Corporation, Doha, Qatar

**Keywords:** natural killer cells, NK receptors, cancer, immunotherapy, immune evasion, checkpoint inhibitors, chimeric antigen receptors, bispecific antibodies

**Editorial on the Research Topic**

**Tailoring NK Cell Receptor–Ligand Interactions: An Art in Evolution**

This research topic is inaugurated by Goh and Huntington, who revise the dynamics of surface receptor expression in murine NK cell subsets at different stages of development (Goh and Huntington). Human NK cell development is subsequently addressed. In their work, Post et al. demonstrate that the transcription factor gene ZNF683/HOBIT is critical for efficient *ex vivo* generation of CD56^+^ NK cells, but likely has limited effects on later acquisition of critical NK cell function modulators, namely NKG2A and killer immunoglobulin-like receptors (KIRs) (Post et al.).

In human disease, *in vivo* selective expansion of phenotypically defined NK cell subsets may affect disease course and response to treatment, a concept underpinned by three manuscripts in this collection. Huenecke et al. report an inverse correlation between the incidence of acute graft-versus-host disease and the frequency of reconstituted CD56 bright NK cells in pediatric patients receiving a hematopoietic stem cell transplantation (HCT) (Huenecke et al.). In their review, Pollmann et al. describe how HCV and human CMV chronic infection affect relative frequency of specific NK cell subsets. The authors specifically revise evidence supporting the concept that genetic background and NK subset composition (e.g., expression of KIR2DL3 in a HLA-C1 homozygous background) promotes HCV clearance and response to treatment (Pollmann et al.). Further elaboration on the importance of NK subpopulation analysis in predicting response to antiviral treatment is provided by Gondois-Rey et al., who report an association between NK maturation phenotype and prompt viremia decrease in response to combination antiretroviral therapy in HIV-infected individuals (Gondois-Rey et al.).

Killer immunoglobulin-like receptor and their interaction with cognate ligands are a major focus of this research topic. Heidenreich and Kröger review the effects of NK cell alloreactivity mediated by inhibitory and activating KIR in unrelated HCT (Heidenreich and Kröger). Erbe et al. analyze the differential impact of alternative HLA-Bw4 antigen groups on the clinical outcome of mAb-based immunotherapy. They previously observed that individuals with follicular lymphoma and neuroblastoma had better clinical outcome following immunotherapy if their HLA/KIR genotypes included *KIR3DL1* and its cognate HLA-Bw4 ligand. The authors now show that this benefit does not extend across all HLA-Bw4 isoforms, but it is only observed for −Bw4 epitopes occurring on HLA-A alleles (HLA-A/Bw4) or HLA-B alleles with Thr amino acid substitution at position 80 (HLA-B/Bw4-T80) (Erbe et al.). Mechanisms of NK tolerance to activating KIR-specific ligands are subsequently addressed in two manuscripts. Carlomagno et al. report that NK cells expressing KIR3DS1 may activate upon recognition of a −Bw4 I80^+^ HLA-B ligand (i.e., HLA-B*51 with Ile at position 80) only if NK donor is −Bw4 I80^−^, thus ensuring tolerance to the self-antigen (Carlomagno et al.). van der Ploeg et al. show that target cell infection with human CMV may potentiate KIR2DS1-mediated positive signaling *in vitro*, suggesting temporary breach of immunological tolerance to self-HLA-C2 in the presence of altered-self (van der Ploeg et al.). Finally, Maniangou et al. describe a novel next-generation sequencing technology for KIR haplotype-wide polymorphism detection, a fast and reliable tool for future studies addressing the effect of KIR allelic diversity in physiology and disease (Maniangou et al.).

Accumulating evidence indicates that positive signaling transduced by NK cell-activating receptors is subject to remarkably complex regulation involving gene expression, ligand interactions, and downstream pathways. Several contributions discuss recent insights into the mechanisms underlying NK cell activation plasticity. NKG2D activating receptor and corresponding ligands are first addressed in a series of focused review articles. Isernhagen et al. address a hot single-nucleotide polymorphism of the MICA NKG2D-binding protein (rs1051792), resulting in a Val129Met substitution. Functional implications of low-affinity 129Met and high-affinity 129Val MICA isoforms on NKG2D-mediated activation are discussed (Isernhagen et al.). Next, Mandelboim and Schmiedel illustrate mechanisms of NKG2D ligand downregulation as a strategy of herpesvirus evasion from NK-mediated immunosurveillance (Schmiedel and Mandelboim). The role of NKG2D and MICA on the outcome of kidney transplantation is revised by Risti and Bicalho. Killer lectin-like heterodimer signaling is addressed next by Pupuleku et al., who utilized a reporter cell system to identify CD94/NKG2C-specific ligands on human CMV-infected cells (Pupuleku et al.). It is increasingly appreciated that ligand diversity and receptor alternative splice variants may potentially result in opposite (i.e., activating versus inhibitory) natural cytotoxicity receptor signaling. In their work, Pazina et al. discuss these phenomena and their potential implications in human physiology and disease (Pazina et al.).

The next section of this research topic describes strategies to enhance the cytotoxicity of cultured NK cells for adoptive immunotherapy. Granzin et al. provide a summary of methods known to promote antitumor reactivity of cultured NK cells and discuss technical and regulatory aspects relevant to NK-based cellular therapy (Granzin et al.). Three studies subsequently address the impact of specific soluble cytokines, cytokine combinations, and feeder cells on NK cell *in vitro* propagation. Sánchez-Correa et al. describe NKp30-specific upregulation and functional reversal of AML-NK cells following short term *in vitro* IL-15 exposure (Sanchez-Correa et al.). Next, Wagner et al. describe a novel NK cell culture protocol based on a two-phase sequential incubation with IL-15 (NK cell expansion) and IL-21 (NK cell functional boost). By using a rhabdomyosarcoma xenogeneic model, the authors show that this protocol may drive propagation of NK cells potentially synergizing radiotherapy antitumor effects (Wagner et al.). Delso-Vallejo et al. focus on the use of irradiated autologous PBMCs as feeders for NK cell culture. This study shows that both feeder–NK physical contact and soluble factors are required for efficient NK cell expansion. Of interest, it also identifies differential transcriptome signatures for proliferating and non-proliferating NK cells (Delso-Vallejo et al.). Strategies to increase sensitivity of tumor cells to NK-mediated lysis are also addressed. Fischer et al. show that incubation with the SMAC mimetic BV6, a selective antagonist of inhibitor of apoptosis proteins, sensitize rhabdomyosarcoma cell lines to NK-mediated killing (Fischer et al.). Moreover, Aquino-López et al. describe the effect of IFNγ on the expression of NK-specific ligands in a panel of tumor cell lines representing variable types of pediatric malignancies. Rationale for these studies derives from the observation that NK cells cultured in the presence of IL-15 and IL-21 secrete high levels of IFNγ upon target recognition, potentially affecting susceptibility to NK lysis (Aquino-López et al.).

Multiple clinical studies have demonstrated the safety and feasibility of allogeneic peripheral blood or cord blood NK cell adoptive immunotherapy. The potential of adoptively transferred allogeneic NK cells as a universal cell therapeutic platform in the transplant and non-transplant settings is addressed by Veluchamy et al. (Veluchamy et al.). An overview of the potential clinical applications of cord blood-derived NK cells is subsequently provided by Sarvaria et al. Tumor immune escape from NK-mediated immunosurveillance may be prevented by redirecting specificity of NK cell effectors. To this end, chimeric antigen receptor (CAR)-modified NK cells engaging tumor-associated antigens have been developed and currently represent a promising approach for clinical translation. Oberschmidt et al. address primary human CAR NK cells as an “off-the-shelf immunotherapy” and describe CAR signaling in NK cells (Oberschmidt et al.). In addition, Zhang et al. review good manufacturing practice-compliant procedures for CAR-engineered NK-92 cells redirected against ErbB2 (HER2) and other tumor epitopes (Zhang et al.). Specific antigen targeting can also be efficiently attained by cross-linking NK cells to cancer cells. In an additional manuscript, Veluchamy et al. demonstrate that lytic activity of cord blood-derived NK cells toward EGFR^+^ colon and cervical cancer cells is strongly enhanced by the mAb cetuximab (Veluchamy et al.). Kloess et al. show that an increased NK cell cytotoxicity leading to B-cell precursor leukemia elimination can be achieved by dual-specific targeting *via* the trispecific immunoligand ULBP2-aCD19-aCD33 (Kloess et al.). Further information on NK-specific dual targeting with triple-specific antibodies to prevent escape of antigen loss variants is provided by Vyas et al. Subsequently, Messaoudene et al. address the potential of NK-based therapy as a tool to enhance potency and prolong efficacy of novel antitumor strategies (Messaoudene et al.). In a specular manner, contemporary therapeutic interventions have the potential to counter tumor-induced NK cell immunosuppression. These effects are covered by Pittari et al., who specifically address the role of NK cells in the context of multiple myeloma (Pittari et al.). To date, preclinical evaluation of NK cell-based therapies in mouse models are challenged by the inherent problem that reagents designed to trigger human immune cells do not react with murine NK cells and by the fact that human NK cell infusions in mice do not provide a human immune cell compartment. Here, Lopez-Lastra and Di Santo describe a Flt3-deficient mouse model allowing for specific enhancement of human NK hematopoiesis *via* exogenous human Flt3 ligand-mediated dendritic cell expansion (Lopez-Lastra and Di Santo). Finally, Hofer and Koehl report some future NK cell-based strategies developed in the context of the European Union ITN NATURIMMUN network and published ahead in *Frontiers in Immunology* (Hofer and Koehl).

## Author Contributions

UK, AT, and GP conceived, designed, and critically revised the manuscript. UK and GP wrote the manuscript. All authors approved the final version of the manuscript.

## Conflict of Interest Statement

The authors declare that the research was conducted in the absence of any commercial or financial relationships that could be construed as a potential conflict of interest.

